# Acceptability of a Chlamydia Vaccine Among Young Women and Men in Birmingham, Alabama, USA: 2023–2025

**DOI:** 10.1093/ofid/ofag166

**Published:** 2026-03-19

**Authors:** Kanupriya Gupta, Bria I Gamble, Madison J D’Amico, Aamuktha Pentala, Muhan Hu, Rebecca J Shiry, Kristal J Aaron, Janeen L Arbuckle, Samantha V Hill, Barbara Van Der Pol, William M Geisler

**Affiliations:** Department of Medicine; University of Alabama at Birmingham, Birmingham, Alabama, USA; Heersink School of Medicine; University of Alabama at Birmingham, Birmingham, Alabama, USA; Department of Medicine; University of Alabama at Birmingham, Birmingham, Alabama, USA; Department of Medicine; University of Alabama at Birmingham, Birmingham, Alabama, USA; Department of Obstetrics and Gynecology; University of Alabama at Birmingham, Birmingham, Alabama, USA; Department of Medicine; University of Alabama at Birmingham, Birmingham, Alabama, USA; Department of Medicine; University of Alabama at Birmingham, Birmingham, Alabama, USA; Department of Obstetrics and Gynecology; University of Alabama at Birmingham, Birmingham, Alabama, USA; Department of Pediatrics; University of Alabama at Birmingham, Birmingham, Alabama, USA; Department of Medicine; University of Alabama at Birmingham, Birmingham, Alabama, USA; Department of Medicine; University of Alabama at Birmingham, Birmingham, Alabama, USA

**Keywords:** chlamydia, STI, vaccine acceptability

## Abstract

**Background:**

Chlamydia infection remains a global health concern, justifying the need for a chlamydia vaccine. Chlamydia infection rates are highest among young women and men; thus, they would be the target populations for vaccination. However, vaccine availability does not ensure uptake. We conducted a survey among youth to identify factors that may influence chlamydia vaccine acceptability.

**Methods:**

Females and males aged 16–29 years seen for care in adolescent, gynecology, student health, and sexual health clinics completed a computer-assisted survey, collecting information on sexual health, health care utilization, vaccination history, and factors related to chlamydia and vaccine acceptability.

**Results:**

Of 399 female and 221 male participants, most (93.2%) had heard of chlamydia, 28.1% reported prior chlamydia infection, and 52.6% believed they could acquire chlamydia infection. Interest in chlamydia vaccination was reported by 42.4% females and associated with non-Black race, Hispanic ethnicity, clinic site, previous influenza or COVID-19 vaccination, and awareness of human papillomavirus vaccination. Among males, 44.8% reported chlamydia vaccination interest that was associated with ethnicity, human papillomavirus vaccine awareness, chlamydia awareness, prior chlamydia infection, and possibility of acquiring chlamydia infection. Among all participants, most (82.5%) reported their main motivation for vaccination was to protect themselves and major barriers were concerns about safety (50.0%) and cost (23.1%). The majority (66.0%) reported health care providers as the main resource for vaccine information and were more likely to get vaccinated if recommended by them.

**Conclusions:**

Less than half of surveyed youth were interested in receiving a chlamydia vaccine. Findings will guide strategies for chlamydia vaccine implementation.

## Teaser

Less than half of surveyed young women and men were interested in receiving a chlamydia vaccine. Main motivation for vaccination was to protect themselves and major barriers were concerns about safety and cost.

Chlamydia infection, caused by *Chlamydia trachomatis* (CT), remains the most commonly reported bacterial sexually transmitted infection (STI) worldwide, despite currently recommended chlamydia prevention and control efforts [1–3]. One factor driving high chlamydia infection rates is the asymptomatic nature of the infection, which leads to many cases being missed and thus continued transmission of CT to others. Another factor is that among treated chlamydia infection cases, reinfection rates are high [4, 5], which also contributes to CT transmission. Our current chlamydia prevention and control efforts aim to identify and treat chlamydia infection in a timely fashion to limit chlamydia-associated complications and continued CT transmission. However, chlamydia treatment early in infection may “arrest” development of protective immunity, as suggested by both animal and human data [6], which could also be a contributing factor for continuation of high chlamydia infection rates. The lack of success of current control strategies all highlight the need for a chlamydia vaccine. Availability of a chlamydia vaccine could have substantial public health benefits, not only in decreasing CT transmission in a population, but also in preventing chlamydia-associated reproductive and perinatal complications [7]. Although currently there are no approved chlamydia vaccines for public use, several promising vaccine candidates are under investigation, including a CT Major Outer Membrane Protein vaccine that was found to be immunogenic in a phase I clinical trial [8–10]. Furthermore, chlamydia vaccines have been included in the World Health Organization's recently published global research priorities for STIs [11].

As learned from human papillomavirus (HPV) vaccine and SARS-CoV-2 (COVID-19) vaccine implementation efforts, availability of a vaccine does not ensure utilization [12, 13]. Vaccine uptake can be impacted by numerous personal health, psychosocial, and political factors, many that are not directly related to sexual health, including: health seeking behaviors; health care access; cultural practices; attitudes of family, friends, and providers; beliefs about vaccine efficacy, safety, and other attributes (eg, cost, delivery mode); and sociopolitical considerations [14–17]. From an STI vaccine acceptability perspective, studies are sparse and used data collected before the COVID-19 pandemic [18]. Recent literature has indicated growth in vaccine hesitancy for pediatric and adult vaccinations in the wake of the COVID-19 pandemic [13, 19]. As such, prior studies may overestimate current vaccine acceptability. Plotnikoff et al and de Waal et al conducted surveys on acceptability of bacterial STI vaccines among Canadians and found a high level of acceptance toward a potential chlamydia vaccine, 74% and 80.3%, respectively [20–22]. A chlamydia vaccine survey study done in adolescent and young adults showed a strong willingness (95%) to receive a chlamydia vaccine; however, the study population had been recently diagnosed with pelvic inflammatory disease and analyses of patient-specific specific factors impacting chlamydia vaccine acceptability were limited [23]. Thus, it is critical that chlamydia vaccine development efforts include further studies aimed at understanding factors, enabling and limiting, that impact vaccine uptake. Such information will be useful in addressing cultural, social, and economic barriers to vaccination and will help guide vaccination education strategies to ensure successful implementation of future chlamydia vaccines. The objective of this study was to better understand the perspectives of young women and men toward a chlamydia vaccine and to identify specific factors that may affect acceptability of a chlamydia vaccine to help guide future education efforts that will improve chlamydia vaccine implementation.

## METHODS

### Setting and Population

The survey was offered to females and males (based on sex assigned at birth) who were presenting for routine care at 4 clinic sites in Birmingham, Alabama: the University of Alabama at Birmingham (UAB) Obstetrics and Gynecology Clinic, the UAB Student Health and Wellness Center, the Children's of Alabama Adolescent Health Center, and the Jefferson County Department of Health Sexual Health Clinic. Eligible patients were defined as those between the ages of 16 and 29 years, not pregnant, and proficient in English. This age range was chosen because it reflects the population with the highest chlamydia infection rates [2]. These clinic sites were chosen to enroll populations who may be representative of those eligible for a future chlamydia vaccine. Predetermined sample size to be enrolled was up to 100 males and 100 females at each clinical site, with the exception of the gynecology clinic, where only females were to be enrolled.

### Procedures

In a private setting at the clinic sites, patients were recruited for the survey study by research staff, and those who agreed to participate provided written informed consent. At the enrollment visit, participants were provided with a computer tablet to complete a self-administered 37-question survey that primarily focused on opinions and beliefs related to chlamydia infection and a future chlamydia vaccine. Participants were free to ask clarifying questions at any time during the survey; however, research staff did not provide input on how to answer the questions. The survey took about 10 minutes to complete, and participants received compensation after completing the survey. Survey responses were collected and stored in a secure, Health Insurance Portability and Accountability Act–compliant Qualtrics (Qualtrics, Provo, UT) account provided by the UAB Information Technology Program.

### Survey Items

The topics and themes addressed in the survey were in part derived from earlier semistructured interviews based on Andersen's Behavioral Model of Healthcare Utilization [24] that were conducted in adolescent health care providers and parents of adolescents in Alabama [25, 26] and from a previously published survey study on interest in bacterial STI vaccines performed at STI clinics in British Columbia [22]. The current survey included sociodemographic information, sexual history, past vaccination knowledge and acceptance, health care provider information, assessment of risk factors for chlamydia infection, interest in chlamydia vaccination, and factors that may affect vaccination interest. We assessed interest in chlamydia vaccination using a Likert scale in which participants could select “very interested,” “interested,” “neutral,” “not interested,” or “very uninterested” in chlamydia vaccination. Participants were limited to choosing 1 main motivation and 1 main barrier of the provided options in the survey. The full survey is available in Supplementary Figure 1.

### Statistics

Descriptive statistics were calculated for all collected data, total, and stratified by female versus male. Chlamydia vaccine acceptability was dichotomized for analysis purposes into 2 categories by participants' response to the question on level of interest in receiving a chlamydia vaccine: “very interested” or “interested” responses were categorized as interested in receiving a chlamydia vaccine, whereas “neutral,” “not interested,” or “very uninterested” responses were categorized as not interested because participants did not express interest (select an interest response to the survey question) in receiving a chlamydia vaccine at the time of survey completion. Sociodemographic characteristics, sexual history, health care utilization, and vaccine history, as well as knowledge, attitudes, and behaviors related to chlamydia infection, were assessed for univariate association with chlamydia vaccination interest using *t*-test or analysis of variance for continuous variables and Fisher exact or chi-squared tests for categorical variables; subanalyses stratified by female versus male were also done. A multivariable conditional logistic regression analysis, matched on sex assigned at birth, which included factors significantly associated with vaccination interest in univariate analyses was done to assess those factors most strongly associated with interest in vaccination in all participants. All analyses were performed using SAS (version 9.4; Cary, NC, USA). A *P* value <.05 was considered statistically significant.

## RESULTS

### Participant Characteristics

The survey was completed between May 2023 and February 2025 by a total of 620 participants (399 females and 221 males) at the 4 clinic sites as follows: UAB Obstetrics and Gynecology Clinic, 100 females; the UAB Student Health and Wellness Center, 100 females and 100 males; the Children's of Alabama Adolescent Health Center, 99 females and 21 males; and the Jefferson County Department of Health Sexual Health Clinic, 100 females and 100 males. Median age of participants was 22 years (range, 16–29 years), with 53.9% (n = 334) reporting Black race, 30.8% (n = 191) White race, 15.3% (n = 95) other races, and 6.5% (n = 40) Hispanic ethnicity (Table 1). Having a regular health care provider was reported by 65.7% (n = 407) participants, and 85.5% (n = 530) reported having health insurance. Majority of participants reported having previous sex (83.4%). A previous STI diagnosis was reported by 34.2% (n = 517), with 28.1% (n = 174) reporting prior chlamydia infection. Majority reported receiving childhood vaccinations (91.6%; n = 568), influenza vaccination (80.8%; n = 501), and COVID-19 vaccination (72.4%; n = 436). Less than half (46.1%; n = 178) reported receiving HPV vaccination.

**Table 1.** Study Participant Characteristics

Characteristic	Total n = 620	Female n = 399 (64.3%)	Male n = 221 (35.7%)	*P* Value
**Sociodemographic**				
Median age, years (range)	22 (16–29)	22 (16–29)	22 (16–29)	.157
Race				<.001
Black	334 (53.9)	211 (52.9)	123 (55.7)	
White	191 (30.8)	144 (36.1)	47 (21.3)	
Other	95 (15.3)	44 (11.0)	51 (23.1)	
Ethnicity^a^				
Hispanic	40 (6.5)	27 (6.8)	13 (5.9)	.001
Non-Hispanic	552 (89.6)	361 (91.4)	191 (86.4)	
Declined to respond	24 (3.9)	7 (1.8)	17 (7.7)	
Level of education^b^				.995
High school	298 (48.2)	192 (48.1)	106 (48.4)	
College	233 (37.7)	151 (37.8)	82 (37.4)	
Postgraduate	87 (14.1)	56 (14.0)	31 (14.2)	
**Clinical**				
Clinic enrolled				<.001
Adolescent health	120 (19.4)	99 (24.8)	21 (9.5)	
Obstetrics and gynecology	100 (16.1)	100 (25.1)	0 (0.0)	
Student health	200 (32.3)	100 (25.1)	100 (45.3)	
Sexual health	200 (32.3)	100 (25.1)	100 (45.3)	
Regular health care provider				<.001
Yes	407 (65.7)	300 (75.2)	107 (48.4)	
No	181 (29.2)	88 (22.1)	93 (42.1)	
Don’t know/not sure	32 (5.2)	11 (2.8)	21 (9.5)	
Health insurance				<.001
Yes	530 (85.5)	363 (91.0)	167 (75.6)	
No	66 (10.7)	25 (6.3)	41 (18.6)	
Don’t know/not sure	24 (3.9)	11 (2.8)	13 (5.9)	
**Sexual health**				
Reports having previous sex				.183
Yes	517 (83.4)	338 (84.7)	179 (81.0)	
No	102 (16.5)	61 (15.3)	41 (18.6)	
Don’t know/not sure	1 (0.2)	0 (0.0)	1 (0.5)	
Reports previous STI diagnosis				.667
Yes	212 (34.2)	134 (33.6)	78 (35.3)	
No	408 (65.8)	265 (66.4)	143 (64.7)	
Clinic participant goes to for sexual and reproductive health care^c^				<.001
Primary care provider	110 (18.0)	60 (15.2)	49 (22.7)	
Obstetrics and gynecology	157 (25.7)	157 (39.8)	0 (0.0)	
Adolescent health	70 (11.4)	61 (15.4)	10 (4.6)	
Student health	70 (11.4)	35 (8.9)	35 (16.1)	
Sexual health	134 (21.9)	58 (14.7)	76 (35.0)	
Walk-in clinic or emergency department	51 (8.3)	20 (5.1)	31 (14.3)	
Other	8 (1.3)	1 (0.3)	7 (3.2)	
Does not have one	12 (2.0)	3 (0.8)	9 (4.2)	
Ever diagnosed with chlamydia infection				.458
Yes	174 (28.1)	108 (27.1)	66 (29.9)	
No	446 (71.9)	291 (72.9)	155 (70.1)	
**Vaccination**				
Received routine childhood vaccinations^d^				
Yes	568 (91.6)	377 (94.5)	191 (86.4)	
No	37 (6.0)	15 (3.8)	22 (10.0)	
Ever had the influenza vaccination				.041
Yes	501 (80.8)	332 (83.2)	169 (76.3)	
No	119 (19.2)	67 (16.8)	52 (23.5)	
Has the influenza vaccination annually^e^				<.001
Yes	254 (50.7)	187 (56.3)	67 (39.6)	
No	247 (49.3)	145 (43.7)	102 (60.4)	
Received 1 or more COVID-19 vaccine doses^e,f^				.017
Yes	436 (72.4)	295 (75.6)	141 (66.5)	
No	166 (27.6)	95 (24.4)	71 (34.5)	
Received 1 or more HPV vaccine doses				<.001
Yes	178 (46.1)	149 (51.2)	29 (30.5)	
No	107 (27.7)	67 (23.0)	40 (42.1)	
Doesn’t know/unsure	101 (26.2)	75 (25.8)	26 (27.4)	

Abbreviations: HPV, human papilloma virus; STI, sexually transmitted infection.

^a^Response missing N = 4 (0.65%).

^b^Response missing N = 2 (0.32%).

^c^Response missing N = 8 (1.29%).

^d^Removed those that selected “Doesn’t know/not sure” (total n = 15, <2.4%).

^e^Missing those who said no on the previous category.

^f^Missing N = 18 (2.9%).

### General Beliefs and Perceptions Toward Chlamydia Infection and a Chlamydia Vaccine

Most participants (93.2%; n = 578) had heard of chlamydia, and 52.6% (n = 326) thought they could get chlamydia infection (Table 2). Overall, 59.4% (n = 368) participants believed that getting a chlamydia vaccine would reduce their risk of getting a chlamydia infection. The majority of participants thought that chlamydia vaccination is an effective way to prevent from getting chlamydia infection (77.3%; n = 479). Among female participants, the majority reported they would receive the chlamydia vaccine to avoid pregnancy complications from chlamydia infection (63.4%; n = 253) and to prevent transmission to their fetus (67.4%; n = 269). Most (90.7%) believed that people should be allowed to get a chlamydia vaccine, if they so choose. The majority (62.9%; n = 390) agreed that they would encourage their partner to get a vaccine, if available.

**Table 2.** General Beliefs and Perceptions Toward Chlamydia Infection and a Chlamydia Trachomatis Vaccine

Characteristic	Total n = 620	Female n = 399 (64.3%)	Male n = 221 (35.7%)	*P* Value
Heard of chlamydia	**…**			.019
Yes	578 (93.2)	379 (95.0)	199 (90.1)	
No	42 (6.8)	20 (5.0)	22 (10.0)	
Think you could get chlamydia infection	**…**			.047
Yes	326 (52.6)	197 (49.4)	129 (58.4)	
No	199 (32.1)	132 (33.1)	67 (30.3)	
Doesn’t know/not sure	95 (15.3)	70 (17.5)	25 (11.3)	
Would get vaccine to reduce risk of getting chlamydia infection	**…**			.762
Agree	368 (59.4)	241 (60.4)	127 (57.5)	
Neutral	176 (28.4)	111 (27.8)	65 (29.4)	
Disagree	76 (12.3)	47 (11.8)	29 (13.1)	
Less likely to use condoms if chlamydia vaccine available	**…**			.010
Agree	74 (11.9)	37 (9.3)	37 (16.7)	
Neutral	122 (19.7)	75 (18.8)	47 (21.3)	
Disagree	424 (68.4)	287 (71.9)	137 (62.0)	
People allowed to get a chlamydia vaccine if they choose to	**…**			.304
Agree	562 (90.7)	367 (92.0)	195 (88.2)	
Neutral	43 (6.9)	21 (6.0)	19 (8.6)	
Disagree	15 (2.4)	8 (2.0)	7 (3.2)	
If received a chlamydia vaccine, would still get STI testing	**…**			.005
Agree	504 (81.3)	339 (85.0)	165 (74.7)	
Neutral	79 (12.7)	39 (9.8)	40 (18.1)	
Disagree	37 (6.0)	21 (5.3)	16 (7.2)	
Chlamydia vaccine is an effective way to prevent chlamydia infection	**…**			.258
Agree	479 (77.3)	316 (79.2)	163 (73.8)	
Neutral	112 (18.1)	65 (16.3)	47 (21.3)	
Disagree	29 (4.7)	18 (4.5)	11 (5.0)	
If chlamydia vaccine available, would encourage partners to get it	**…**			.294
Agree	390 (62.9)	252 (63.2)	138 (62.4)	
Neutral	169 (27.3)	113 (28.3)	56 (25.3)	
Disagree	61 (9.8)	34 (8.5)	27 (12.2)	
Would get immunized to avoid pregnancy complications^a^	**…**			
Agree	253 (63.4)	253 (63.4)		
Neutral	109 (27.3)	109 (27.3)	-	-
Disagree	37 (9.3)	37 (9.3)		
Would get immunized to avoid transmission to their fetus^a^	**…**			
Agree	269 (67.4)	269 (67.4)		
Neutral	98 (24.6)	98 (24.6)	-	-
Disagree	32 (8.0)	32 (8.0)		

Abbreviation: STI, sexually transmitted infection.

^a^Not applicable to males; total percentages calculated from n = 399.

### Chlamydia Vaccine Interest and Vaccine Interest Motivators and Barriers

**Figure 1.**
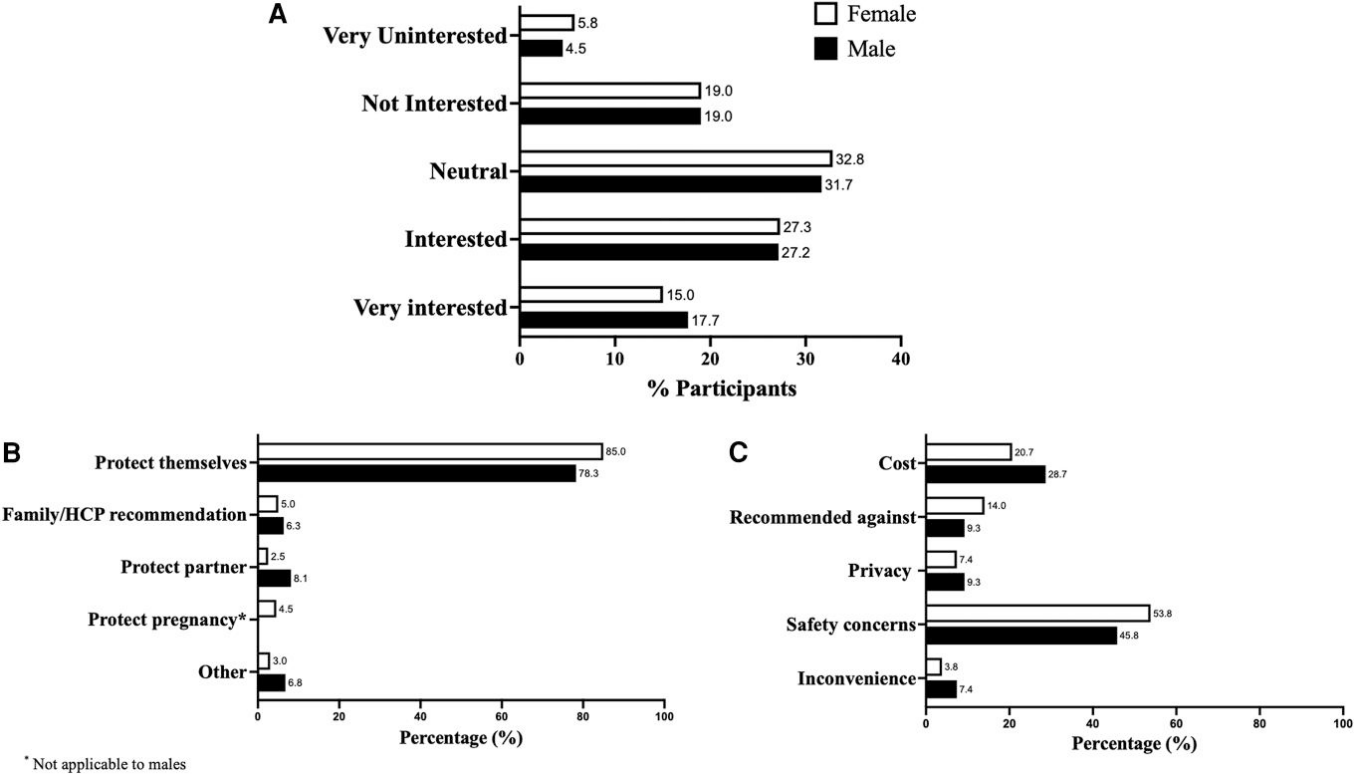
Chlamydia vaccine acceptability and associated motivators and barriers in females and males. *A*, Bar graph depicting the distribution of chlamydia vaccine acceptability based on a Likert scale in which participants could select “very interested,” “interested,” “neutral,” “not interested,” or “very uninterested” in receiving a chlamydia vaccine in females (n = 399) and males (n = 221). *B*, Bar graph depicting the distribution of main motivator to receive a chlamydia vaccine in females (n = 399) and males (n = 221). *C*, Bar graph depicting the distribution of main barrier to not receive a chlamydia vaccine in females (n = 392) and males (n = 216). Data missing for <2% participants (n = 12).

Less than half of participants, 42.4% (n = 169) females and 44.8% (n = 99) males, were interested in receiving a chlamydia vaccine, if available (Figure 1*A*). The remaining were “neutral” (females, 32.8% [n = 131]; males, 31.7% [n = 70]), “not interested” (females, 19.0% [n = 76]; males, 19.0% [n = 42]), or “very uninterested” (females, 5.8% [n = 23]; males, 4.5% [n = 10]) in receiving a chlamydia vaccine.

Primary motivation for receiving a chlamydia vaccine among all participants was to protect themselves (all, 82.7% [n = 512]; females, 85.0% [n = 339]; males, 78.3% [n = 173]) (Figure 1*B*) and main barriers for not receiving a vaccine were safety concerns (all, 50.0% [n = 310]; females, 53.8% [n = 211]; males, 45.8% [n = 99]), followed by cost (all, 23.1% [n = 143]; females, 20.7% [n = 81]; males, 28.7% [n = 62]) (Figure 1*C*); the main barrier significantly differed (*P* < .001) based on vaccine interest, with those interested in receiving a chlamydia vaccine more often reporting cost as the main barrier compared to those not interested (31.7% vs 17.3%), whereas those not interested more often reported safety concerns (62.4% vs 35.9%).

### Association of Chlamydia Vaccine Interest With Participant Characteristics, Beliefs, and Perceptions

Among all participants, interest in receiving a chlamydia vaccine was associated with non-Black race (*P* = .011), Hispanic ethnicity (*P* = .002), diagnosis of prior chlamydia infection (*P* = .033), awareness of chlamydia infection (*P* = .003), perceived risk for chlamydia infection (*P* = .006), history of vaccination for influenza (*P* = .010) and COVID-19 (*P* = .006), receiving influenza vaccination annually (*P* = .013), HPV vaccine awareness (*P* < .001), and receiving HPV vaccination (*P* = .019) (Table 3). For females, interest in receiving a chlamydia vaccine was associated with non-Black race (*P* = .024), Hispanic ethnicity (*P* = .035), site of enrollment (*P* = .016), history of vaccination for influenza (*P* = .005) and COVID-19 (*P* < .001), receiving influenza vaccination annually (*P* = .047), and HPV vaccine awareness (*P* = .007). For males, vaccine interest was associated with Hispanic ethnicity (*P* = .023), awareness of chlamydia infection (*P* = .008), perceived risk for chlamydia infection (*P* = .015), diagnosis of prior chlamydia infection (*P* = .028), and awareness of HPV vaccine (*P* = .010). Because there were differences in participant characteristics associated with vaccine interest based on sex assigned at birth, we did further subanalyses to investigate this. We found that women more often reported having received vaccinations for influenza (*P* < .001), COVID-19 (*P* = .017), and HPV (*P* < .001) compared to men. Men more often perceived having risk for getting chlamydia infection compared to women (*P* = .047). On multivariable analysis, the following factors remained significantly associated with chlamydia vaccination interest among all participants: non-Black race (*P* = .009), Hispanic ethnicity (*P* = .011), diagnosis of prior chlamydia infection (*P* < .001), awareness of chlamydia infection (*P* = .018), perceived risk for chlamydia infection (*P* = .008), history of COVID-19 vaccination (*P* = .014), and HPV vaccine awareness (*P* = .012).

**Table 3.** Association of Vaccine Acceptability With Participating Characteristics, Beliefs, and Perceptions Among All Participants (n = 620)

Characteristic	Total			Female			Male		
	Interested, n = 268 (43.2%)	Not Interested, n = 352 (56.8%)	*P* Value	Interested, n = 169 (42.4%)	Not Interested, n = 230 (57.6%)	*P* Value	Interested, n = 99 (44.8%)	Not Interested, n = 122 (55.2%)	*P* Value
**Sociodemographic**									
Age, median (range)	22 (16–29)	22 (16–29)	.678	21 (16–29)	22 (16–29)	.651	23 (16–29)	22 (16–29)	.165
Race			.011			.024			.135
Black	128 (47.8)	206 (58.5)		76 (45.0)	135 (58.7)		52 (52.5)	71 (58.2)	
White	99 (36.9)	92 (26.1)		72 (42.6)	72 (31.3)		27 (27.3)	20 (16.4)	
Other	41 (15.3)	54 (15.3)		21 (12.4)	23 (10.0)		20 (20.2)	31 (25.4)	
Ethnicity^a^			.002			.035			.023
Hispanic	27 (10.1)	13 (3.7)		17 (10.1)	10 (4.6)		10 (10.8)	3 (2.7)	
Non-Hispanic	235 (87.7)	317 (91.1)		152 (89.9)	209 (95.4)		83 (89.3)	108 (97.3)	
Level of education^b^			.157			.196			.496
High school	120 (44.8)	178 (50.9)		73 (43.2)	119 (51.7)		47 (47.5)	59 (49.2)	
College	103 (38.4)	130 (37.1)		68 (40.2)	83 (36.1)		35 (35.4)	47 (39.2)	
Postgraduate	45 (16.8)	42 (12.0)		28 (16.6)	28 (12.2)		17 (17.2)	14 (11.7)	
**Clinical**									
Clinic enrolled			.085			.016			.886
Adolescent health	46 (17.2)	74 (21.0)		38 (22.5)	61 (26.5)		8 (8.1)	12 (9.9)	
Obstetrics and gynecology	39 (14.6)	61 (17.3)		39 (23.1)	61 (26.5)		45 (45.5)	55 (45.5)	
Student health	101 (37.7)	99 (28.1)		56 (33.1)	44 (19.1)		46 (46.5)	54 (44.6)	
Sexual health	82 (30.6)	118 (33.5)		36 (21.3)	64 (27.8)				
Regular health care provider			.790			.709			.240
Yes	178 (66.4)	229 (65.1)		126 (74.6)	174 (75.7)		52 (52.5)	55 (45.1)	
No	78 (29.1)	103 (29.3)		37 (21.9)	51 (22.2)		41 (41.4)	52 (42.6)	
Doesn’t know/unsure	12 (4.5)	20 (5.7)		6 (3.6)	5 (2.2)		6 (6.1)	15 (12.3)	
Health insurance^c^			.058			.309			.154
Yes	224 (83.6)	306 (86.9)		151 (92.1)	212 (94.6)		73 (76.0)	94 (83.9)	
No	36 (13.4)	30 (8.5)		13 (7.9)	12 (5.4)		23 (24.0)	18 (16.1)	
**Sexual health**									
Reports having sex before^d^			.178			.280			.394
Yes	230 (85.8)	287 (81.5)		147 (87.0)	191 (83.0)		83 (83.8)	96 (79.3)	
No	38 (14.2)	64 (18.2)		22 (13.0)	39 (17.0)		16 (16.2)	25 (20.7)	
Reports previous STI diagnosis			.277			.790			.152
Yes	98 (36.6)	114 (32.4)		58 (34.3)	76 (33.0)		40 (40.4)	38 (31.2)	
No	170 (63.4)	238 (67.6)		111 (65.7)	154 (67.0)		59 (59.6)	84 (68.9)	
Clinic participant goes to for sexual and reproductive health care^e^			.337			.050			.110
Primary care provider	45 (17.0)	65 (18.7)		21 (12.5)	40 (17.6)		24 (24.7)	25 (20.8)	
Obstetrics and gynecology	66 (24.9)	91 (26.2)		66 (39.3)	91 (40.1)		0 (0.0)	1 (0.8)	
Adolescent health	30 (11.3)	41 (11.8)		25 (14.9)	35 (15.4)		5 (5.2)	5 (4.2)	
Student health	38 (14.3)	32 (9.2)		22 (13.1)	13 (5.7)		16 (16.5)	19 (16.0)	
Sexual health	54 (20.4)	79 (22.8)		20 (11.9)	38 (16.7)		34 (35.1)	42 (35.0)	
Walk-in clinic or emergency department	27 (10.2)	24 (6.9)		10 (6.0)	10 (4.4)		17 (17.5)	14 (11.7)	
Other	2 (0.8)	6 (1.7)		1 (0.6)	0 (0.0)		1 (1.0)	6 (5.0)	
Does not have one	3 (1.1)	9 (2.6)		3 (1.8)	0 (0.0)		0 (0.0)	9 (7.5)	
Ever diagnosed with chlamydia infection			.033			.332			.028
Yes	87 (32.5)	87 (24.7)		50 (29.6)	58 (25.2)		37 (37.4)	29 (23.8)	
No	181 (67.5)	265 (75.3)		119 (70.4)	172 (74.8)		62 (62.6)	93 (76.2)	
Heard of chlamydia infection			.003			.107			.008
Yes	259 (96.6)	319 (90.6)		164 (97.0)	215 (93.5)		95 (96.0)	104 (85.3)	
No	9 (3.4)	33 (9.4)		5 (3.0)	15 (6.5)		4 (4.0)	18 (14.8)	
Think you could get chlamydia infection			.006			.084			.015
Yes	154 (57.5)	171 (48.6)		86 (50.9)	111 (48.3)		68 (68.7)	61 (50.0)	
No	68 (25.4)	132 (37.5)		47 (27.8)	85 (37.0)		21 (21.2)	46 (37.7)	
Doesn’t know/unsure	46 (17.2)	49 (13.9)		36 (21.3)	34 (14.8)		10 (10.1)	15 (12.3)	
**Vaccination**									
Received routine childhood vaccinations^f^			.273			.597			.361
Yes	252 (94.0)	316 (89.8)		163 (97.0)	214 (95.5)		89 (91.8)	102 (87.9)	
No	13 (4.9)	24 (6.8)		5 (3.0)	10 (4.5)		8 (8.3)	14 (12.1)	
Ever had the influenza vaccine			.010			.005			.464
Yes	229 (85.5)	272 (77.3)		151 (89.4)	181 (78.7)		78 (78.8)	91 (74.6)	
No	39 (14.6)	80 (22.7)		18 (10.7)	49 (21.3)		21 (21.2)	31 (25.4)	
Has the influenza vaccine annually^g^			.013			.047			.109
Yes	130 (56.8)	124 (45.6)		94 (62.3)	93 (51.4)		36 (46.2)	31 (34.1)	
No	99 (43.2)	148 (54.4)		57 (37.8)	88 (48.6)		42 (53.9)	60 (65.9)	
Heard of the COVID-19 vaccines			.556			.177			.734
Yes	259 (96.6)	343 (97.4)		163 (96.5)	227 (98.7)		96 (97.0)	116 (95.1)	
No	9 (3.4)	9 (2.6)		6 (3.6)	3 (1.3)		3 (3.0)	6 (4.9)	
Received 1 or more COVID-19 vaccine doses^g,h^			.006			<.001			.965
Yes	202 (78.0)	233 (67.9)		138 (84.7)	157 (69.2)		64 (66.7)	77 (66.4)	
No	57 (22.0)	110 (32.1)		25 (15.3)	70 (30.8)		32 (33.3)	39 (33.6)	
Heard of the HPV vaccine			<.001			.007			.010
Yes	187 (69.8)	200 (56.8)		135 (79.9)	156 (67.8)		52 (52.5)	43 (35.3)	
No	81 (30.2)	152 (43.2)		34 (20.1)	74 (32.2)		47 (47.5)	79 (64.8)	
Received 1 or more HPV vaccine doses^g^			.019			.061			.066
Yes	98 (52.4)	80 (40.0)		77 (57.0)	72 (46.2)		21 (40.4)	8 (18.6)	
No	41 (21.9)	67 (33.5)		23 (17.0)	44 (28.2)		18 (34.6)	22 (51.2)	
Doesn’t know/unsure	48 (25.7)	53 (26.5)		35 (25.9)	40 (25.6)		13 (25.0)	13 (30.2)	

Abbreviations: HPV, human papilloma virus; STI, sexually transmitted infections.

^a^Missing N = 4 (0.65%); removed those that selected “decline to answer” (total n = 24, 3.87%).

^b^Missing N = 2 (0.32%).

^c^Removed those that selected “Doesn’t know/not sure’ (Total n = 24, 3.87%).

^d^Removed 1 (0.16%) response of “Doesn’t know/not sure.”

^e^Missing N = 8 (1.29%).

^f^Removed those that selected “Doesn’t know/not sure’ (total n = 15, 2.42%).

^g^Missing those who said no on the previous category.

^h^Missing N = 18 (2.90%).

### Chlamydia Vaccine Implementation Considerations

The majority of participants (66.0%; n = 409) reported health care providers as their main resource for vaccine information (females, 70.7% [n = 282]; males, 57.5% [n = 127]). Most participants (74.2%; n = 460) agreed that they were more likely to receive a chlamydia vaccine if recommended by a health care provider as compared to a friend (31.9%; n = 198) or a family member (40.8%; n = 253) (Table 4). Compared to participants who did not report interest in receiving a chlamydia vaccine, those who reported an interest in receiving a chlamydia vaccine were significantly more likely to find 2 or more vaccine doses to be acceptable (75.4% vs 46.0%; *P* < .001) and willing to receive a vaccine booster (82.1% vs 40.1%; *P* < .001). Although the majority of participants (62.0%; n = 384) were not willing to pay out of pocket for a chlamydia vaccine, those interested in getting a chlamydia vaccine were significantly more likely to be willing to pay (50.0% vs 28.8%; *P* < .001). The majority of participants (52.7%; n = 326) thought adolescence was the best time to offer the vaccine, and those interested in receiving the vaccine more often thought late childhood or adolescence was the best time compared with those not interested (67.2% vs 56.4%; *P* = .004).

**Table 4.** Chlamydia Vaccine Implementation Considerations Among Participants by Vaccine Interest

Consideration	Total, N = 620	Interested n = 268 (43.2%)	Not Interested n = 352 (56.8%)	*P* Value
Main vaccine information resource				.139
Family or friends	15 (2.4)	2 (0.8)	13 (3.7)	
Health care providers	409 (66.0)	176 (65.7)	233 (66.2)	
Marketing resources	66 (10.7)	29 (10.8)	37 (10.5)	
Trusted organizations	121 (19.5)	58 (21.6)	63 (17.9)	
Other	9 (1.5)	3 (1.1)	6 (1.7)	
More likely to get a chlamydia vaccine if recommended by a friend				<.001
Agree	198 (31.9)	114 (42.5)	84 (23.9)	
Neutral	249 (40.2)	106 (39.6)	143 (40.6)	
Disagree	173 (27.9)	48 (17.9)	125 (35.5)	
More likely to get a chlamydia vaccine if recommended by a family member				<.001
Agree	253 (40.8)	133 (49.6)	120 (34.1)	
Neutral	235 (37.9)	96 (35.8)	139 (39.5)	
Disagree	132 (21.3)	39 (14.6)	93 (26.4)	
More likely to get a chlamydia vaccine if recommended by a health care provider				<.001
Agree	460 (74.2)	241 (89.9)	219 (62.2)	
Neutral	108 (17.4)	19 (7.1)	89 (25.3)	
Disagree	52 (8.4)	8 (3.0)	44 (12.5)	
Number of vaccine doses acceptable^a^				<.001
1	250 (41.1)	66 (24.6)	184 (54.0)	
2	254 (41.7)	122 (45.5)	132 (38.7)	
3	54 (8.9)	42 (15.7)	12 (3.5)	
4	51 (8.4)	38 (14.2)	13 (3.8)	
Willing to get a vaccine booster^b^				<.001
Yes	360 (58.4)	220 (82.1)	140 (40.1)	
No	257 (41.7)	48 (17.9)	209 (59.9)	
Vaccine booster frequency participants found acceptable^c^				.541
Annually	154 (42.9)	99 (45.0)	55 (39.6)	
Once every 2 years	117 (32.6)	70 (31.8)	47 (33.8)	
Once every 5 years	71 (19.8)	43 (19.6)	28 (20.1)	
Once every 10 years	17 (4.7)	8 (3.6)	9 (6.5)	
Participant willing to pay out of pocket for a chlamydia vaccine^d^				<.001
Yes	235 (38.0)	134 (50.0)	101 (28.8)	
No	384 (62.0)	134 (50.0)	250 (71.2)	
When participants think is the best time to offer a chlamydia vaccine^d^				.004
Late childhood (9–12 years old)	52 (8.4)	31 (11.6)	21 (6.0)	
Adolescence (13–17 years old)	326 (52.7)	149 (55.6)	177 (50.4)	
Adulthood (≥18 years old)	241 (38.9)	88 (32.8)	153 (43.6)	

^a^Missing n = 11 (1.8%).

^b^Missing n = 3 (0.05%).

^c^Missing n = 4 (0.065%) and those that said they were not willing to get a vaccine booster.

^d^Missing n = 1 (0.016%).

## DISCUSSION

The success of a chlamydia vaccine program will not only depend on a vaccine's efficacy in preventing chlamydia infection, persisting infection, and associated complications, but also on the willingness of the target populations to take the vaccine. Our study assessed the acceptability of a future chlamydia vaccine among a diverse population of adolescents and young adults, aiming to identify key factors influencing willingness to receive chlamydia vaccination. Our main finding that less than half of young women and men were interested in receiving a chlamydia vaccine follows a concerning trend reported in other recent studies on STI vaccine acceptability [18]. This generally unfavorable perspective toward a chlamydia vaccine is a significant shift from earlier studies that evaluated chlamydia vaccine acceptability, in which most of the surveys were done pre–COVID-19 and were framed in the context of STI prevention [20–23]. HPV vaccine studies have also reported lower vaccine acceptability post–COVID-19 [12]. Despite instances where the majority of our survey participants reported perceiving certain benefits of receiving a chlamydia vaccine, such as reducing risk of getting chlamydia infection and preventing transmission to fetus, reservations or concerns about the vaccine itself may have hindered them from being willing to receive it even though they could perceive a benefit. There has been a growing mistrust toward vaccination since the COVID-19 pandemic [27]. We found that concern about vaccine safety was the most commonly selected reason for disinterest in the chlamydia vaccine. A subset also considered perceived cost of the future vaccine as a significant barrier. Uncertainty about insurance coverage could also contribute to general mistrust and hesitancy, although this was not assessed in the survey. Overall, public skepticism toward a novel vaccine might result in low chlamydia vaccine uptake, regardless of published safety or efficacy data.

To improve chlamydia vaccine acceptability, targeted public health messaging about chlamydia infection and the importance of being vaccinated to prevent it and its complications is crucial. We examined participant characteristics that may impact vaccine acceptability and found that demographic variables influenced acceptability. Those reporting Black race were less often interested in vaccination versus those reporting White or other races, and those reporting Hispanic ethnicity were more often interested in vaccination compared with those reporting as non-Hispanic; given the higher chlamydia infection rates in the United States among those reporting Black race compared with other reported races, this is a concerning finding [2]. Although frequency of chlamydia vaccination interest was similar in females and males, there were clear differences in factors that impacted chlamydia vaccine acceptability between females versus males, suggesting biologic sex-specific messaging may be an important consideration in chlamydia vaccine education efforts. Unlike in some previous studies, we found that males perceived higher risk of getting chlamydia infection than females [28]. Although vaccine acceptability was significantly associated with perceived susceptibility to chlamydia overall, the finding was only significant in males. Although we found that women had higher rates of influenza, HPV, and childhood vaccinations, lower interest in chlamydia vaccination provided an interesting dichotomy that might be influenced by stigma, perceived sexual activity, or underestimating their personal risk for chlamydia infection, particularly if they are monogamous or have the belief that STI screening is sufficient. We found that the most commonly selected motivating factor for receiving a chlamydia vaccine was to protect oneself, as has also been reported in previous studies by Plotnikoff et al and de Waal et al, where participants' desire to protect themselves and their partners were the most important factors driving chlamydia vaccine acceptability [20, 22]. Together, these findings support a need for targeted approaches to chlamydia vaccine education, which will likely play a pivotal role in increasing chlamydia vaccine uptake.

Health care provider recommendation emerged as a crucial factor in vaccine decision-making. Most participants reported health care providers as the main resource for vaccine information and indicated a strong likelihood of accepting the vaccine if recommended by a health care provider as compared to family and friends. Provider recommendation has consistently been shown to be the strongest predictor of HPV vaccine uptake [29] and a potential chlamydia vaccine [30], underscoring the role of provider–patient communication in promoting vaccine confidence. Normalizing the vaccine as part of routine preventive care and delivering strong, clear recommendations could significantly improve acceptance among both females and males. Simplicity of a chlamydia vaccine regimen, in terms of number of doses and need for a booster, may also be an important factor influencing acceptability of a chlamydia vaccine. The majority of those who did not express interest in receiving a vaccine reported the maximum vaccine doses they were willing to receive was one and the majority were not willing to receive a booster. In contrast, the majority of those who were interested in receiving a chlamydia vaccine were willing to receive two or more doses and a booster.

Although our survey study was the first to evaluate chlamydia vaccine acceptability after the post–COVID-19 pandemic and focused solely on a chlamydia vaccine (not done in the context of other STI vaccines), generalizability of the findings may be limited in that this survey was carried out in populations seen at clinics that provide sexual health care and who live in an urban metropolitan area in the Southeastern United States. Such factors could impact the knowledge of and prior experience with chlamydia infection and its associated complications as well as with vaccines. Because a chlamydia vaccine is currently unavailable, study participants could possibly have had difficulty conceptualizing vaccine considerations for a theoretical vaccine. Study participants also completed the survey on a computer tablet while in the clinic and it is possible that could have impacted their responses compared to if they had completed it in a more private, personal setting. The study had a higher proportion of female participants, which may introduce bias. Additionally, some data were excluded because of missing survey responses. Our survey did not explore if chlamydia vaccine acceptability would differ based on whether the vaccine was an mRNA-based versus non-mRNA based formulation, which may be important to study further given perceived and real safety concerns that were seen with COVID-19 mRNA vaccines [31]. Additional chlamydia vaccine acceptability studies that capture community-based or general populations in other geographical locations and further explore perceptions about mRNA-based chlamydia vaccines are needed. Also, research on parental perspectives on chlamydia vaccination are sparse [26], and further studies are needed.

In conclusion, our findings indicate a concerning trend of low acceptability for a potential chlamydia vaccine, underscoring significant challenges to future chlamydia vaccine uptake. Clear educational messaging and health care provider endorsement are required to ensure effective chlamydia vaccine uptake. Addressing misconceptions and safety concerns as well as raising awareness of the severity of chlamydia-associated disease will be essential for successful implementation. These insights can inform public health strategies and vaccine rollout plans once a chlamydia vaccine becomes available.

## Supplementary Material

ofag166_Supplementary_Data

## Supplementary Data

Supplementary materials are available at *Open Forum Infectious Diseases* online. Consisting of data provided by the authors to benefit the reader, the posted materials are not copyedited and are the sole responsibility of the authors, so questions or comments should be addressed to the corresponding author.
