# Supplementary material for: Acceptability of a Chlamydia Vaccine Among Young Women and Men in Birmingham, Alabama, USA: 2023–2025
Source: Open Forum Infect Dis. 2026 Mar 19;13(4):ofag166. doi: 10.1093/ofid/ofag166 (PMC13064519; doi:10.1093/ofid/ofag166)
Supplement: ofag166_Supplementary_Data [file ofag166_supplementary_data.pdf]

THE UNIVERSITY OF ALABAMA AT BIRMINGHAM

Background

The purpose of the survey is to understand what factors may influence acceptability of a vaccine against the sexually transmitted infection (STI) chlamydia. Your participation is voluntary and does not affect your clinic care today in any way. Your survey responses will remain anonymous and confidential. All questions are optional. Please do **NOT** write your name on the survey.
First, we would like to collect sociodemographic characteristics from you.

What is your age?

What is your gender identity?

- ○ Cis-female ○ Cis-male ○ Transgender-female ○ Transgender-male
- ○ Non-binary or gender fluid ○ Decline to answer
- ○ Other, please specify:

What is your highest level of education?

- ○ Currently in some level of high school ○ High school graduate/GED
- ○ Some college/Associate's Degree ○ Bachelor's Degree ○ Any post-graduate studies
- ○ Decline to answer

Please select your race (Select one or more as appropriate):

- ☐ White ☐ Black or African American ☐ American Indian or Alaska Native ☐ Asian
- ☐ Native Hawaiian or Pacific Islander ☐ Other, please specify.
- ☐ Decline to answer

Please select your ethnicity:

- Non-Hispanic/Non-Latino
- Hispanic/Latino
- Decline to answer

Next, we would like to ask you about your health care.

	Yes	No	Don't know/Not sure
Do you have a regular health care provider or family doctor?	○	○	○
Do you have health insurance coverage?	○	○	○
Did you receive routine childhood vaccinations when you were growing up?	○	○	○
Did your parents tell you that childhood vaccines were important?	○	○	○

Have you ever received the flu vaccine?

- Yes
- No

Do you get the flu vaccine every year?

- Yes
- No

Have you heard of the COVID-19 vaccine?

- Yes
- No

Have you received one or more doses of the COVID-19 vaccine?

- Yes
- No

If you have received the COVID-19 vaccine, at what age did you receive your first vaccination for COVID-19?

[ ]

If you have received the COVID-19 vaccine, did you receive a booster?

○ Yes ○ No

Next, we would like to ask you about your sexual health.

	Yes	No	Don't know/Not sure
Have you ever had sex before?	○	○	○
Have you heard about sexually transmitted infections (STIs)?	○	○	○
Have you ever been diagnosed with a STI?	○	○	○

Where do you usually go for sexual or reproductive healthcare?

○ STI Clinic ○ OB-Gyn Clinic ○ Adolescent Health Clinic ○ Primary Care Provider
○ Walk-in clinic or ER ○ Student Health
○ Other location, please specify:
[ ]

Have you ever heard of the HPV vaccine?

○ Yes ○ No

Have you received one or more doses of the HPV vaccine?

○ Yes ○ No ○ Not sure/Don't know

How many shots of the HPV vaccine did you receive?

○ 1 ○ 2 ○ 3 ○ Not sure/Don't know

If you did receive the HPV vaccine, at what age did you receive your first vaccination for HPV?

Chlamydia

	Yes	No	Don't know/Not sure
Have you ever heard about chlamydia?	○	○	○
Do you think you could get chlamydia?	○	○	○
Have you ever been diagnosed with chlamydia?	○	○	○

Chlamydia is a common STI that can be treated and cured with antibiotics. Chlamydia does not cause symptoms in the majority of patients, however, in some patients it can cause genital and pelvic symptoms. Regardless of whether chlamydia causes symptoms or not, it can potentially cause infertility, may affect pregnancy outcomes (for example, could cause preterm birth), could be transmitted to a newborn baby during delivery causing infection of the eye or lungs, and can increase a person's risk to get HIV infection if exposed.

Please rate the following statement by your level of interest: "If available today, I would be interested in receiving a vaccine to prevent chlamydia".

○ Very uninterested ○ Not interested ○ Neutral ○ Interested ○ Very interested

Please think about all of your reason(s) that would motivate you to receive a chlamydia vaccine. Please note the MAIN reason (one reason) and then ALL reasons.

	MAIN reason (choose only one)	ANY reason (select any that apply)
a. To protect myself against chlamydia	☐	☐
b. To protect my partner from getting chlamydia	☐	☐
c. Lower the need to treat recurring chlamydia using medication	☐	☐

	MAIN reason (choose only one)	ANY reason (select any that apply)
d. Fewer visits to the doctor or clinic	☐	☐
e. I would use condoms less if I received a chlamydia vaccine	☐	☐
f. Because a friend recommended it	☐	☐
g. Because a family member recommended it	☐	☐
h. Because a health care provider recommended it	☐	☐
i. If pregnant, to prevent against pregnancy complications from chlamydia* (* for women in which the question is applicable)	☐	☐
j. If pregnant, to protect my unborn child from chlamydia* (* for women in which the question is applicable)	☐	☐

Please think about all of your reason(s) that you may **not** be interested in receiving a chlamydia vaccine. Please note the MAIN reason (one reason) and then ALL reasons.

	MAIN reason (choose only one)	ANY reason (select all that apply)
a. Cost of the vaccine	☐	☐
b. Inconvenient setting/location for getting the vaccine	☐	☐
c. Privacy (having the vaccination on my health record)	☐	☐
d. I am unsure of the protection or benefits of the vaccine.	☐	☐
e. I worry about a new chlamydia vaccine being unsafe.	☐	☐
f. I might be judged or feel embarrassed about getting a chlamydia vaccine.	☐	☐
g. Because a friend recommended against it	☐	☐
h. Because a family member recommended against it	☐	☐
i. Because a health care provider did not offer it	☐	☐

Please indicate how strongly you agree or disagree with each of the statements below.

	Strongly disagree	Disagree	Neutral	Agree	Strongly agree
a. I would be less likely to use condoms if a vaccine was available to prevent chlamydia.	○	○	○	○	○
b. People should be allowed to get vaccinated to prevent chlamydia if they choose to.	○	○	○	○	○
c. If available, I would be interested in getting a vaccine to reduce my risk of getting chlamydia.	○	○	○	○	○
d. If I were to receive a vaccine to prevent chlamydia, I would still go to get STI testing.	○	○	○	○	○
e. I think a vaccine could be an effective way to prevent chlamydia.	○	○	○	○	○
f. If a chlamydia vaccine were available, I would encourage my partner(s) to get it.	○	○	○	○	○
g. I would be more likely to get a chlamydia vaccine if it was recommended by a friend.	○	○	○	○	○
h. I would be more likely to get a chlamydia vaccine if it was recommended by a family member.	○	○	○	○	○
i. I would be more likely to get a chlamydia vaccine if it was recommended by a health care provider.	○	○	○	○	○

	Strongly disagree	Disagree	Neutral	Agree	Strongly agree
j. If a chlamydia vaccine were available, I would get immunized to avoid potential pregnancy complications.* (* for women in which the question is applicable)	○	○	○	○	○
k. If a chlamydia vaccine were available, I would get immunized to avoid transmission of chlamydia to my child during birth.* (* for women in which the question is applicable)	○	○	○	○	○

Please think about where you would like to receive information about a chlamydia vaccine if it were available. Please note the MAIN resource and then all additional ways to access information.

	MAIN resource (choose only one)	ANY resources (select all that apply)
a. In person conversations with a physician, nurse, or nurse practitioner	☐	☐
b. Trusted organizations (CDC, NIH, WHO, health department)	☐	☐
c. Trusted family members or friends	☐	☐
d. Brochures in clinics	☐	☐
e. Online resources and social media (ex. Facebook, Twitter, TikTok)	☐	☐
f. Public advertisements (public transit posters and billboards)	☐	☐
g. Other, specify. ________	☐	☐

What is the maximum number of initial chlamydia vaccine doses you would be willing to receive?

○ 1 ○ 2 ○ 3 ○ 4

Would you be willing to get a chlamydia vaccine booster?

○ Yes ○ No

How often would you be willing to get a chlamydia booster?

○ Annually ○ Once every 2 years ○ Once every 5 years ○ Once every 10 years

Would you be willing to pay out of pocket (pay for it yourself) to receive a chlamydia vaccination?

○ Yes ○ No

When do you think it would be best to first offer a chlamydia vaccine, if available?

○ Late childhood (9-12 years old) ○ Adolescence (13-17 years old)
○ Early adulthood (18-30 years old) ○ Adulthood (>30 years old)

Would you be interested in participating in a more in-depth in-person interview on the acceptability of a chlamydia vaccine at a later date ?

○ Yes ○ No

If you are interested in participating in a more in-depth in-person interview at a later date on acceptability of a chlamydia vaccine, please provide a contact phone number.

[ ]

Powered by Qualtrics
